# Supplementary figures and images for: Diet-induced glial insulin resistance impairs the clearance of neuronal debris in Drosophila brain
Source: PLoS Biol. 2023 Nov 7;21(11):e3002359. doi: 10.1371/journal.pbio.3002359 (PMC10629620; doi:10.1371/journal.pbio.3002359)

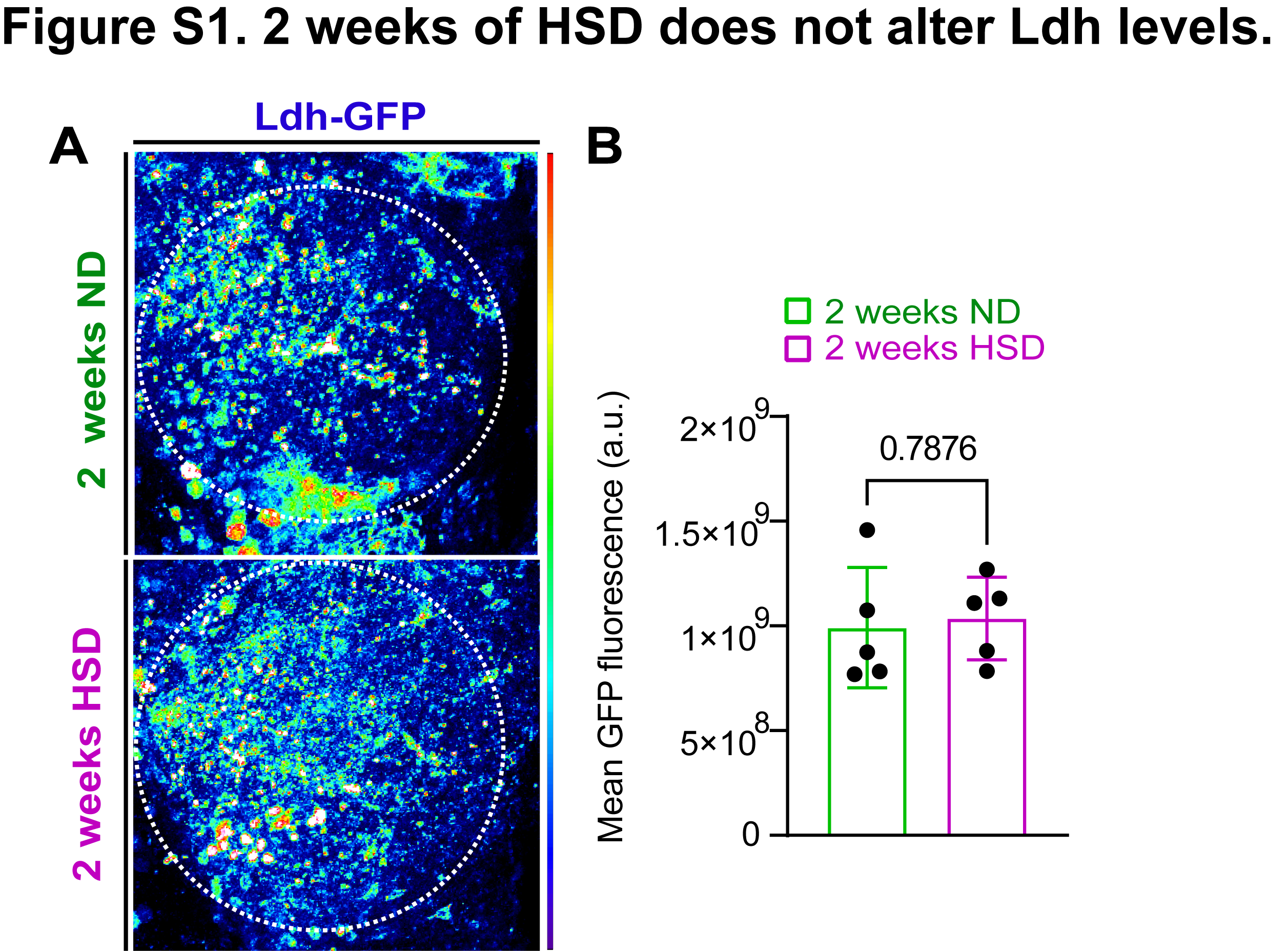

Supplement: S1 Fig — (A) Confocal images of Ldh-GFP in the antennal lobe region (dotted circle) of ND and HSD-fed flies after 2 weeks of diet treatment. (B) Mean Ldh-GFP fluorescent intensity, obtained from Z-stack summation projections, within a defined region of interest (dotted circle) that covers the antennal lobe in ND and HSD-fed flies. The data underlying this figure can be found in the Supporting information file S5 Data. (TIF) [file pbio.3002359.s001.tif]

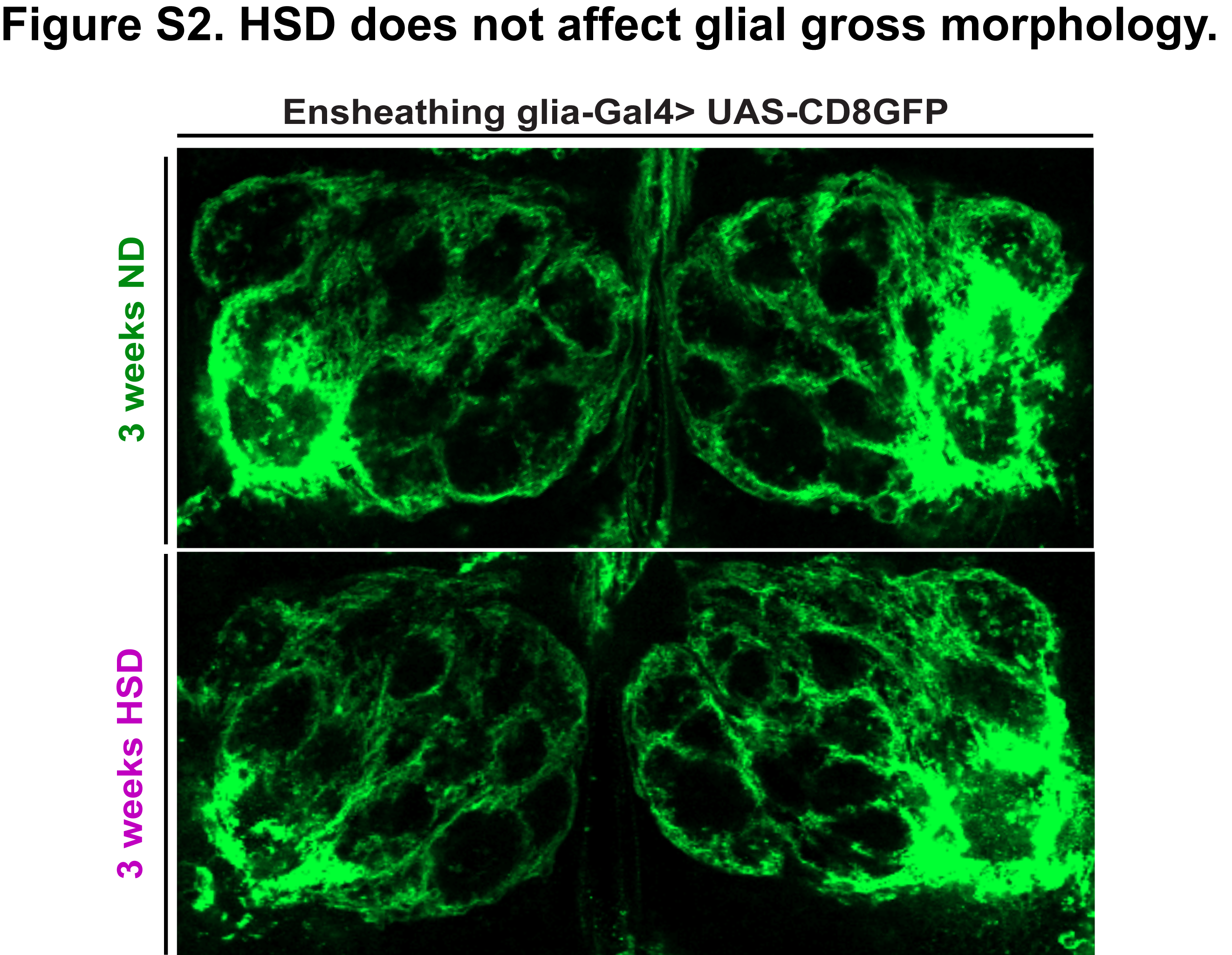

Supplement: S2 Fig — No observable gross morphological defects in the HSD-fed flies. (TIF) [file pbio.3002359.s002.tif]

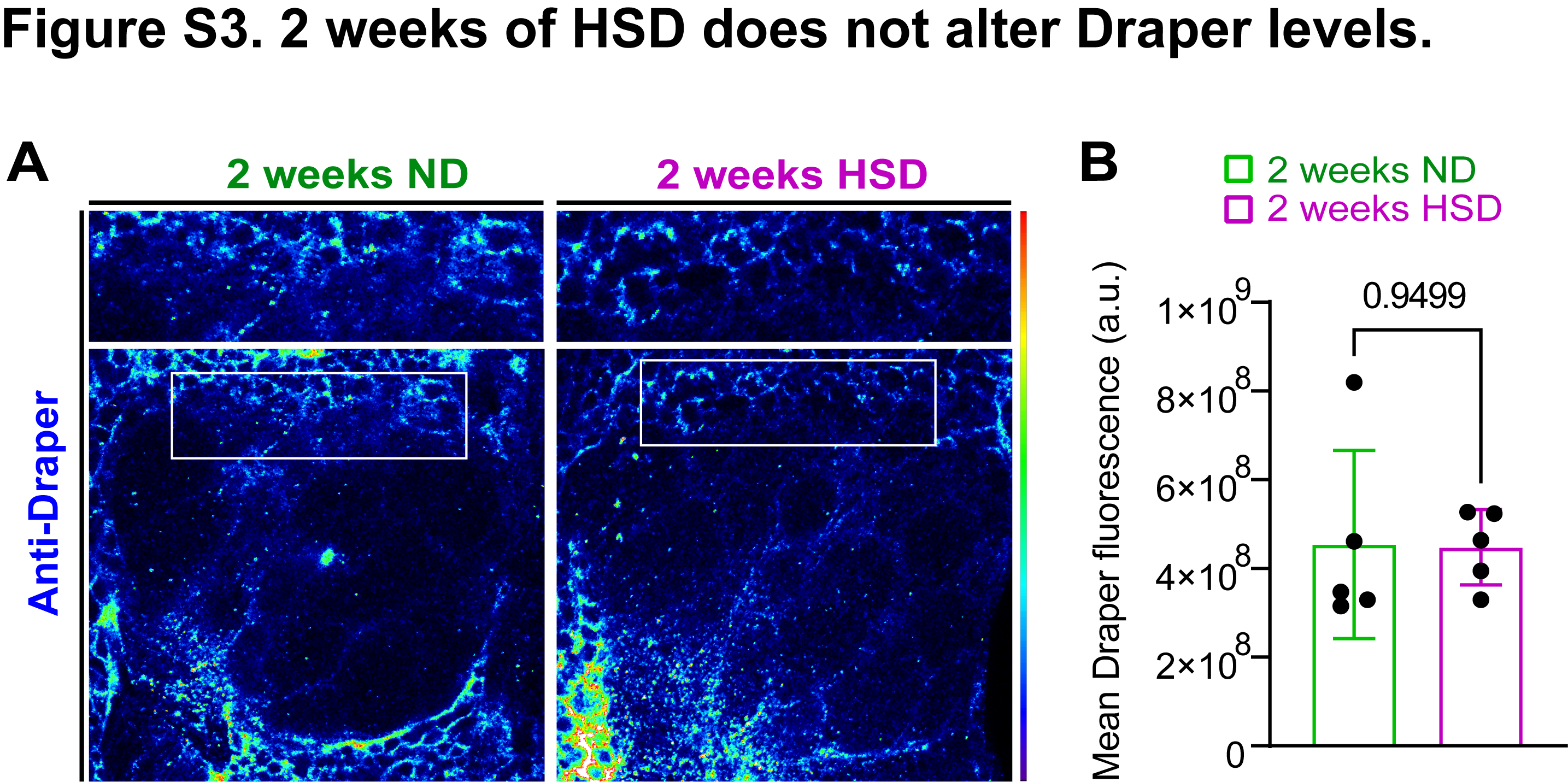

Supplement: S3 Fig — (A) Confocal images of the antennal lobe region of flies fed an ND or an HSD for 2 weeks that were immunostained with anti-Draper. (B) Mean fluorescent intensity of Draper measured within a region of interest (white box) that coincides with the location of ensheathing glia in ND and HSD-fed flies. Measurements were obtained from a Z-stack summation projection that spans the entire depth of the antennal lobe. Student t test with Welch’s correction. N = each circle represents an individual fly. The data underlying this figure can be found in the Supporting information file S6 Data. (TIF) [file pbio.3002359.s003.tif]

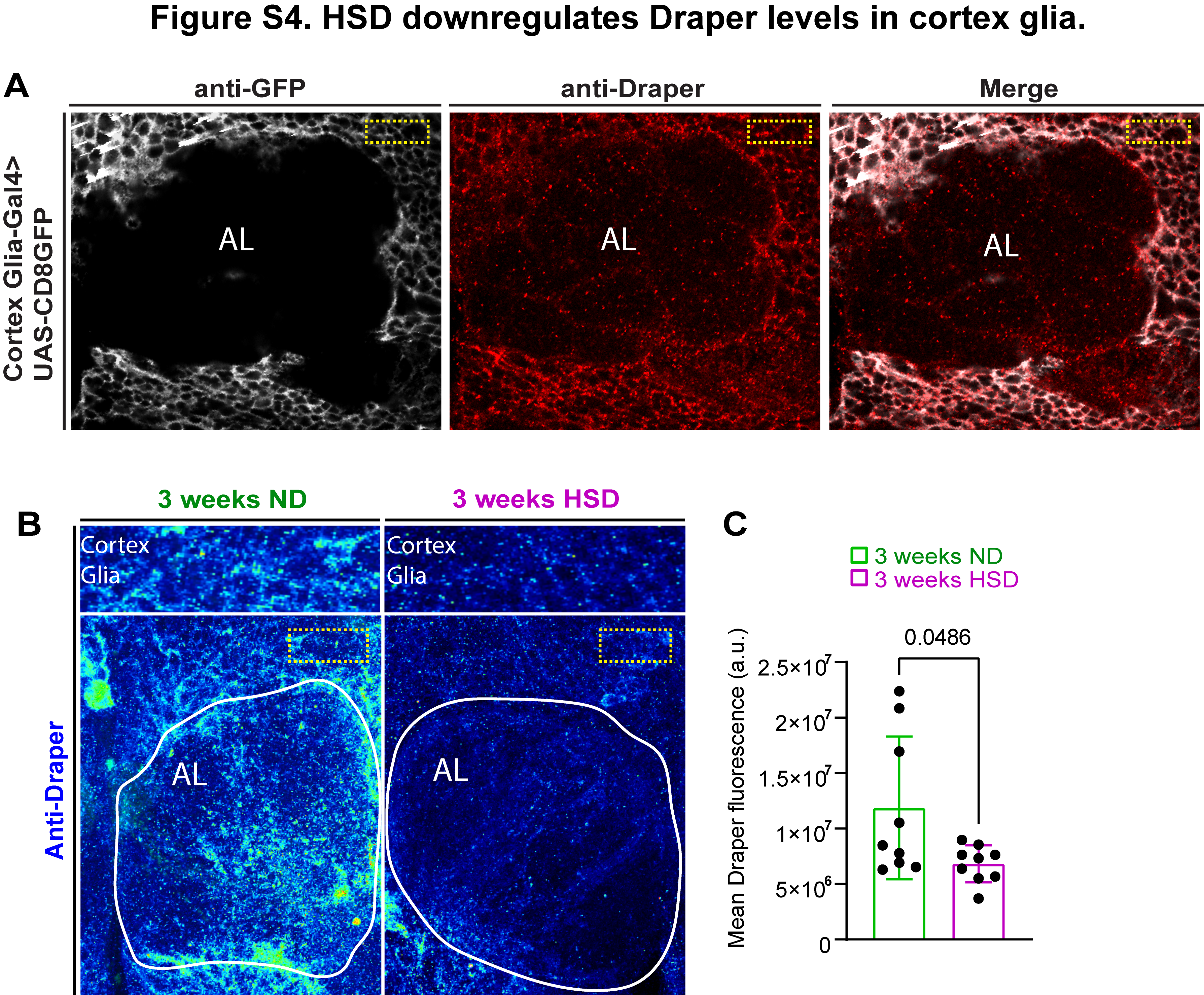

Supplement: S4 Fig — (A) Representative confocal images of the antennal lobe region of flies expressing membrane GFP under the control of a cortex glia-specific Gal4 driver. Draper immunostaining colocalizes with cortex glia. The yellow box represents a sample ROI in which Draper fluorescence was measured. (B) Confocal images of the antennal lobe region of flies fed an ND or an HSD for 2 weeks that were immunostained with anti-Draper. (C) Mean fluorescent intensity of Draper measured within a region of interest (white box) that coincides with the location of cortex glia in ND and HSD-fed flies. Measurements were obtained from a Z-stack summation projection that spans the entire depth of the antennal lobe. Student t test with Welch’s correction. N = each circle represents an individual fly. The data underlying this figure can be found in the Supporting information file S7 Data. (TIF) [file pbio.3002359.s004.tif]
