# Supplementary material for: Diet-induced glial insulin resistance impairs the clearance of neuronal debris in Drosophila brain
Source: PLoS Biol. 2023 Nov 7;21(11):e3002359. doi: 10.1371/journal.pbio.3002359 (PMC10629620; doi:10.1371/journal.pbio.3002359)
Supplement: Attachment — Submitted filename: PLoS Bio rev response_081623_MA.docx [file pbio.3002359.s014.docx]

**Response to Reviewers:**

*We appreciate all the reviewers’ for their thoughtful consideration of our study. We are encouraged by their overall positive assessment of our findings that high-sugar diet regime causes insulin resistance in glia and impairs glial phagocytic function. We are deeply grateful for the constructive feedback and specific suggestions of the two anonymous Reviewers #1, 3 and Dr. Melkani (#2). In the revised version, we have endeavored to enact on this feedback that has improved the clarity and rigor of our work. Please see our point-point response below. In addition, to the point-point response, we refer the reviewers to the last page of this document “revised experiment list” that summarizes all the new experiments that were performed, and the corresponding updated figure panel that will give an overview of all the changes.* **Reviewer #1:** This manuscript examines the interactions between diet and glial debris clearance, using a well-established model of Wallerian degeneration in Drosophila.  Previously research groups have found that this process is regulated by insulin, aging, and sleep.  It is therefore very surprising that the connection to diet has not been investigated.  This line of investigation is potentially highly impactful because of links between dietary dysregulation of insulin and negative public health outcomes.  In addition, the manuscript provides a direct link between high-sugar diet and changes within glial cells.  Therefore, it is of broad interest to researchers studying glial biology, diet and metabolism, and aging.  The experiments are well presented and elegantly straight-forward.  The statistical analyses are appropriate, and the experiments are well-controlled.  The data is presented in a way that is highly accessible to diverse research groups. I have a number of suggestions below.  Given this is a short report they can likely all be addressed with minor text edits. Overall, this work was a pleasure to read and opens up exciting new avenues of investigation for multiple fields of nutrition and neuroscience.

**We thank Reviewer #1 for their fair and balanced assessment and are delighted to note that they believe our study will be of broad interest. We have strived to address their comments to the best of our capacity.

1.      The data in 1L may be improved by quantification, or at the very least higher magnification images.**
We thank the reviewer for their suggestion. We have included the quantification of LipidTox fluorescent intensity in the revised manuscript (Figure 1H). **2.      The Dilp5 results seem to be overinterpreted.  The reduced levels could also be due to reduced synthesis under the conditions, and this needs to be documented at some form (at least at the transcript level).**

We appreciate the reviewer's comment regarding the potential limitations of relying solely on reduced Dilp5 fluorescence as evidence to conclude that HSD increases Dilp5 levels. Per the reviewer’s suggestion, we have included the transcript levels of Dilp5 (Figure 2E) in the revised version of the manuscript. Specifically, we performed qPCR for Dilp5 in the adult fly brain under normal diet and after flies were subjected to a 3-week high-sugar diet (HSD). On HSD steady-state Dilp5 mRNA levels are significantly increased (Figure 2E). As we presented previously, we observed reduced immunofluorescence of Dilp5 in the Insulin Producing Cells (IPCs) on HSD (Figure 2C, 2E). We note this observation that a “rich” diet increases Dilp5 secretion (as assayed by immunofluorescence levels) is consistent with reports from other groups(1, 2). Taken together these results suggest that though HSD increases Dilp5 production (at the transcript level), the reduced Dilp5 protein in the IPCs points to increased secretion.

**3.      The manuscript does not address the specific effects of high sugar diet versus increased caloric intake.  Including this in the discussion, or experimentally, would increase impact.  A few sentences on this matter would add to the impact of the discussion.**

We thank the reviewer for raising this question. Indeed, it is imperative that we provide a clear rationale for choosing a 30% high sugar diet, and not just a high calorie diet. To address this, we have included the following paragraph in the discussion

“*“In our studies, we have adopted a 30% high sugar diet regime. The rationale for this stems from prior studies showing that larvae reared on high-sugar diets develop insulin resistance and diabetic phenotypes. Musselman and colleagues found that isocaloric diets high in protein and fat did not affect insulin signaling (3). According to this study, insulin resistance phenotypes were associated with an increase in sugar intake but not with an increase in calorie intake (3). Consistent with this, we observed peripheral insulin resistance in adult Drosophila raised on a 30% HSD (4). It is worth noting, however, that other obesogenic regimes in Drosophila, such as coconut oil based high fat diets (HFDs), have been used to explore time-restricted feeding's effect on obesogenic decline (14, 17). Given that different obesogenic regimes are likely to have varied impacts, future work will be needed to assess whether a HFD regime causes glial insulin resistance in Drosophila.*”

Furthermore, instigated by this reviewer’s question on rationale, we have added experimental evidence to provide rationale for the 3-week time point in HSD. As we now shown in Figure S1 and Figure S3, the levels of glycolytic enzyme LDH and draper expression in glial cells, is not impacted by 2 weeks of HSD, but only at the 3-week time point. We believe emphasizing the temporal aspect is important, as most studies of HSD regime in *Drosophila* focus on earlier 7-10 days. Whereas impact of HSD on glial insulin resistance and draper expression manifests at a prolonged exposure of 3 weeks. Thus, our studies are likely to stimulate others in the *Drosophila* metabolism researchers to examine phenotypes on HSD later point (> 2 weeks on HSD).

**4.      While discussed in tangential ways, please include a direct comparison of the similarities and differences between ensheathing glia and microglia. In addition, the discussion does not include whether the effects observed are specific to microglia or generalizable to all glial types.**

We have incorporated the following content into the introduction section

“*Glial phagocytosis begins with the recognition of cellular debris via cell-surface receptors. Ablation of these receptors results in impaired clearance of cellular debris, while their overexpression leads to excessive neuronal pruning(3). In Drosophila, a distinct subtype of glial cells known as ensheathing glia serve as the brain’s resident phagocytes. Analogous to mammalian microglia, ensheathing glia respond to neuronal injury by extending their membrane processes towards the site of damage and initiating phagocytic activity. Furthermore, just like their mammalian counterparts, Drosophila ensheathing glia (4), express phagocytic receptors, most prominently the mammalian Multiple EGF Like Domains 10 (MEGF10) homolog, Draper (5). Also akin to microglia, ensheathing glia have shown proficiency in clearing ectopically expressed human amyloid beta (Aβ) via the phagocytic receptor Draper in a Drosophila model of Alzheimer's disease(6).”*

In addition to ensheathing glia, cortex glia also express Draper. Upon further analysis, we found that Draper levels in the cortex glia of HSD-fed flies. This data is now included in the revised manuscript (now Figure S4). We chose to focus on ensheathing glia in this study due to their well-established role in phagocytosis and to take advantage of the experimentally accessible antennal lobe region.

**5.      I have some concern about the use of maximum intensity to quantify images.  I recognize that this is a standard in the field, however, it masks a significant amount of data throughout the Z-stack.  Perhaps a supplemental movie depicting the full Z-stack for critical data sets would increase data accessibility.**

We thank the reviewer for their suggestion. We have included supplemental 3D movies of the olfactory axons in the ND and HSD-fed flies post injury (Figure 4G)**.

___________________________________________________**

**Reviewer #2: *Girish C. Melkani, UAB Heersink of Medicine (note, reviewer 2 has signed this review):* In this short communication, using a Drosophila model, the author shows that a chronic obesogenic diet induces glial insulin resistance and impairs the clearance of neuronal debris by downregulating the glia-associated phagocytic receptor, Draper. Genetically attenuating systemic insulin release rescues diet-induced glial insulin resistance and draper expression, while stimulating Phosphoinositide 3-kinase (PI3K) rescues high-sugar diet-induced glial defects. This study establishes that obesogenic diets impair glial phagocytic function, resulting in delayed clearance of neuronal debris. This is an interesting finding, and we need more studies like this, however, some additional assays will be required to make these findings complete and more impactful. Therefore, the following questions need to be addressed for consideration in PLoS Biology.**

We are thankful to Dr. Melkani for a thoughtful and engaged review of this manuscript. We have executed a new set of functional experiments (Figure 1) to assess whole brain metabolic state. To the extent possible within the scope of this study we have strived to address Dr. Melkani’s comments experimentally and with textual changes. We hope that Dr. Melkani finds this manuscript has improved based on their feedback.

**1.      Many downstream targets of insulin receptors are known, including Stat92E, however, it is unclear why PI3K was selected. The author should confirm the expression of other downstream targets with qPCR.**

The PI3K pathway is a known downstream arm of insulin signaling. Therefore, it has been used as a reliable readout of insulin sensitivity. Nonetheless, we appreciate the reviewer’s question as examining other markers for insulin resistance will indeed strengthen the scientific rigor of our claim. For the revised version, we have tested two more targets that are associated with insulin resistance and find the results to be consistent.

I) *Glucose-6-Phosphate Dehydrogenase (G6P).* It has been shown that G6P levels are inversely correlated with insulin signaling sensitivity (7-10). We found that HSD treatment significantly increases G6P transcript levels indicating insulin resistance (Figure 2A).
II) IRS – Chico: Additionally, we show that the insulin receptor substrate, Chico, is upregulated on HSD (Figure 2B), which normally occurs under low insulin signaling (11) conditions indicating insulin resistance (Figure 2B).

**2.      Other complications linked with obesity have not been discussed have nor other obesogenic conditions.**

We have added the following sentences in the introduction page 1, lines 5-7, to broaden the complications linked with obesity and other non-obesogenic conditions.

“The effects of obesity on organ function are wide-ranging, including improper lipid accumulation in non-adipose tissues such as the muscle, resulting in cardiac dysfunction, and affecting life expectancy (12-14).”

**3.      Obesogenic diet is not limited to HSD, Does the author report a similar phenotype with HFD and/or missed diets (HSD+HFD), which will mimic with human obesogenic diet?**

We appreciate the reviewer’s comment that there are other obesogenic diets in *Drosophila* including HFD (5% coconut oil) and HFD+ HSD (5% coconut oil + 300mM sucrose). We have noted that Dr. Melkani’s group performs comprehensive studies using this diet regime on time restricted feeding (TRF) on muscle function(12, 15). However, despite our efforts to use a coconut oil-based feeding regime, we are unable to obtain flies that survive on these diets beyond 10 days. This is likely due to humidity, basal recipe, and housing conditions differences between our labs.

We find that a 30% HSD regime is most suited to study the impact of prolonged exposure to HSD(16). Hence, for this study we have specifically used the same 30% HSD regime we had previously established as the most robust, thereby allowing us to assess the effect of prolonged HSD. *Critically, central to the thesis of this study, we find that the 30% HSD regime diminishes insulin sensitivity in glia (Figure 2); and we showed in our previous study(16) that 30% HSD regime causes peripheral insulin resistance.* Given that our focus is on what happens to glia when brain insulin levels are changed due to diet or genetic manipulations, we are confident that the 30% HSD regime is sufficient to address the goal we set out to study. Nonetheless, we have included that provides a clear rationale for this HSD- regime experimental design, including a note about HFD diets (see underlined sentence).

*“In our studies, we have adopted a 30% high sugar diet regime. The rationale for this stems from prior studies showing that larvae reared on high-sugar diets develop insulin resistance and diabetic phenotypes. Musselman and colleagues found that isocaloric diets high in protein and fat did not affect insulin signaling(17). According to this study, insulin resistance phenotypes were associated with an increase in sugar intake but not with an increase in calorie intake(17). Consistent with this, we observed peripheral insulin resistance in adult Drosophila raised on a 30% HSD(16). It is worth noting, however, that other obesogenic regimes in Drosophila, such as coconut oil based high fat diets (HFDs), have been used to explore time-restricted feeding's effect on obesogenic decline (12, 15). Given that different obesogenic regimes are likely to have varied impacts, future work will be needed to assess whether a HFD regime causes glial insulin resistance in Drosophila.*

”

**4.      The author has indicated that microglia are the brain's resident macrophages, and they play a key role in learning and memory. However, no attempt is made to test memory impairment linked with HSD and other genetic modulations the authors have made this question. It is unclear what makers are used for neuroinflammation.**

Dr. Melkani’s question likely arises from the following sentences in the introduction:

“A key feature of neurodegenerative disorders is the diminished clearance of neuronal debris and neuron-secreted toxic proteins. *This can lead to inflammation*, secondary neuronal death, and impaired axonal regeneration. Microglia are the brain’s resident macrophages, *and they play a key role in learning and memory*.”

Our goal in the introduction was to set up a broad role for microglia in brain function which includes learning and memory. However, we would like to clarify that the scope of our study is primarily focused on investigating whether prolonged HSD causes glial insulin resistance. If it does, can it impair one well established function of ensheathing glia- phagocytosis? We appreciate the reviewer's suggestion to explore the effects of HSD-induced obesity on learning and memory, but it is outside the scope and capabilities of our lab. Nonetheless, we are in conversation with groups that specialize in learning and memory, and hope our conversations lead to a productive collaboration on this aspect of how HSD affects cognitive function. In the revised version, we have removed the *purple underlined part of the sentence in the introduction* as it has caused an expectation that learning, and memory is an aspect of this present study.

**5.      I am unclear how the authors rule out the first source of energy balance, i.e., glycolysis in Fig1. Multiple findings showed that this can also be a source of energy balance.**

Prompted by the reviewer’s question, *we’ve included a new set of functional experiments* for which we adopted methods from Marla Tipping’s group(18) that measure whole brain metabolism. In these experiments, we measured the basal Extracellular Acidification Rate (ECAR), a readout of glycolysis of adult fly brains subject to HSD (Figure 1B-D). Combined with the reduced expression of the Ldh reporter, our results suggest that prolonged HSD treatment attenuates whole brain glycolysis possibly via downregulating Ldh.

**6.      Mitochondrial morphology alteration in the fat tissue in revealing in the brain function is not convincing for mitochondrial function, as their shape is unchanged in the brain (fig. 1 E, F). Is there interorgan communication from fat tissue to the brain? If yes, this must be addressed.**

We agree with the reviewer that mitochondrial morphology readout in the brain we presented in the original version was not convincing. Given this concern, we have removed those cell-imaging based indirect measures. In the revised manuscript we present a direct measure of OxPhos using Oxygen consumption rate (OCR) of the whole adult fly brain using the seahorse flux analyzer (Figure 1B). We have included the following sentence in the results section.

*“We found that the brains of HSD-treated flies exhibited an elevation in OCR (Figure 1B) and a concomitant reduction in ECAR (Figure 1C), indicating a clear shift from glycolysis towards OxPhos.”*

This study focuses on whether high-sugar regimes result in glial insulin resistance. Perhaps the reviewer is referring to a new and ongoing area of investigation in our lab that is yet to come to fruition and is not a part of this work.

**7.      Fig. 1K is convincing, however, quantification will be required, which is lacking.**

We thank the reviewer for his feedback. We have now included quantification of the LipidTOX fluorescent intensity (Figure 1H).

**8.      It is unclear in Figs. 2-4, what basis rectangles were selected for data analyses? Not clarified what area of the brain is used for quantification. It seems biased approach, and it must clarify, including a detailed description in the method section.**

We greatly appreciate the reviewer's feedback. We have adapted the same methods taken by leaders in the field(19-27). In response to their suggestions, we have provided a more detailed description of our methods. Specifically, we have included the following information in the methods: “*Ldh fluorescent intensity was measured within an ROI that covered the entirety of the left antennal lobe. Draper, Stat92 and PI3K fluorescent intensity were performed within an ROI in the dorsal medial antennal lobe membrane as previously described(19, 23, 27). Cortex glia’s Draper fluorescent intensity was measured by drawing a region of interest above the antennal lobe (See representative ROI in Figure S4). The size and dimensions of all ROIs were maintained consistently throughout each experiment*.”

**9.      Overall, this interesting finding is limited to cytological/genetic analyses, and these data along with the behavior/functional analyses will make this more impactful and will be suitable for publication in PLoS Biology.**

We would like to address the reviewer's comment by highlighting the significance of quantifying axonal debris as a well-established functional measure for evaluating glial phagocytosis. Also, taking the reviewer’s feedback on improving our functional assays, we set up the *ex-vivo* metabolic brain measurements to assess the metabolic impact of 3-week HSD on the brain (Figure 1B-D). As we mention in comment #4 above, behavioral assays on learning and memory are beyond the scope and capabilities of our lab. Nonetheless, we conclude by thanking the reviewer for suggesting that we broaden our horizons on the cognitive aspects of HSD impact on glial function.

**Additional comments:
1.      Upon chronic exposure to an HSD, circulating insulin levels rise, resulting in insulin resistance----------------------------it should be circulating sugar levels rise, correct.**

Yes. That is correct. But in the revised version, we also show results that insulin transcripts increase while the Dilp5 levels in IPCs reduce (Figure 2C-E).

**2.      Insulin resistance linked with obesogenic challenges has been tested in Drosophila peripheral organs, however, this was not discussed.**

We have referenced our previous study(16) which shows that the same chronic HSD treatment results in insulin resistance in fat tissue. This was determined by using the nuclear localization of the forkheadbox O (FOXO) (Kelly et al., 2022; Figure 2B), which has been reliably used as a proxy to monitor insulin sensitivity.

**3.      The figure legends and method sections are brief and do not provide sufficient information. These should be addressed in the revised version.** 
We appreciate the reviewer’s concerns and we have made changes to address them in the revised manuscript.

**Reviewer #3: The manuscript by Alassaf and Rajan elegantly reveals that insulin resistance is induced in the adult Drosophila brain by a chronic high sugar diet (HSD). The authors nicely show that this diet delays clearance of injured axon debris by ensheathing glia as a result of reduced levels of the phagocytic receptor Draper (basal and injury-induced).

This interesting and very well written manuscript exposes a possible connection between obesogenic diets and impaired clearance of neuronal debris in adult brain, which might be implicated in understanding the pathophysiology of neurodegenerative diseases. The findings are novel and significant for a wide research community.**

**We thank reviewer#3 for their positive assessment and strived to address the issues raised below.**

**1. The authors focus on the specific type of glia, ensheathing glia, which was shown to perform phagocytosis of axonal debris. Since neurodegeneration can involve different mechanisms from injury-induced wound healing mechanism, other types of glia might be also involved. Cortex and astrocyte-like glia, which can be also phagocytic were previously shown to response differently to Insulin signaling ("Independent glial subtypes delay development and extend healthy lifespan upon reduced insulin-PI3K signaling" by Nathaniel S. Woodling et al., 2020). It is interesting to know whether HSD-induced or systemic insulin resistance in other types of glia also affect Draper expression in these cells.**

While we acknowledge the potential involvement of other glial subtypes, such as astrocytes, in the olfactory bulb and their role in wound healing, a previous study by Doherty et al. (2009) (26) have established that astrocytes do not exhibit morphological changes in response to axonal damage, nor do they express the phagocytic receptor, Draper. In contrast, cortex glia do express Draper, and in our revised manuscript we show that cortex glia of HSD-fed flies have reduced Draper levels (Figure S4). However, for the purpose of this study, we chose to concentrate on ensheathing glia, leveraging the well-established axonal injury model of the olfactory lobe.

**2. In Figure 1L statistics of accumulation of lipid droplets is missing. Do other types of glia also accumulate lipid droplets? In some cases, accumulation of lipid droplets can be neuroprotective ("Antioxidant Role for Lipid Droplets in a Stem Cell Niche of Drosophila" by Andrew P Bailey et al., 2015). It is worth to discuss it.**

We thank the reviewer for their comment. We have included quantification of LipidTox fluorescent intensity in the revised manuscript (Figure 1H). We have included a sentence in the results section saying the following to keep open the possibility raised by Alex Gould’s group that Lipid Droplets can be neuroprotective in the appropriate context.

*“Though Lipid Droplets can be neuroprotective in the appropriate context(28), Lipid droplet accumulation and reduced glycolysis are tightly associated with impaired insulin signaling [25, 43-46].”*

**3. What happens to mitochondria in neurons? Do they look differently on HSD and under normal conditions?**

In the original manuscript we used mitochondrial morphology as a readout for Oxidative Phosphorylation. But given the questions raised about it being an indirect measure, we have now chosen to remove the cell-based imaging morphometrics and instead used a functional assay to test this. In the revised manuscript, we present direct measure of OxPhos using Oxygen consumption rate (OCR) measurements of the whole adult fly brain using the seahorse flux analyzer under HSD (Figure 1B). We have included the following sentence in the results section.

*“We found that the brains of HSD-treated flies exhibited an elevation in OCR (Figure 1B) and a concomitant reduction in ECAR (Figure 1C), indicating a clear shift from glycolysis towards OxPhos.”*

**Minor comments:**
- All gene names and genotypes must be in italics everywhere including Figures and Fly husbandry, no capital letters (page 5 "draper gene"), protein names must start with a Capital letter (Draper - line 13 in the abstract)
- LipidTOX is written differently in different places
- Please remove pointing to Figures from Discussion
- In "image analysis" there is "A" in the middle of the sentence: "To measure mitochondrial morphology, a maximum…"

We have addressed these minor comments. Thank you for the detail-oriented corrections!

**Revised Experiments List.**

***Figure 1B:*** HSD treatment stimulates mitochondrial OxPhos in the brain. This data addresses the following comments:

- **Reviewer 2**
  - In comment #6, reviewer 2 has stated that mitochondrial morphology alone is not sufficient to infer mitochondrial function. We performed *ex-vivo* whole brain metabolic analysis to measure mitochondrial oxygen consumption rate (OCR), a direct readout of mitochondrial oxidative phosphorylation (Figure 1B).
  - In comment #9, reviewer 2 has raised concerns that the manuscript relies mainly on cytological approaches. In the revised manuscript, we performed a sophisticated *ex vivo* whole brain metabolic analysis using Agilent Seahorse (Figure 1B-C).
- **Reviewer 3**
  - In comment #3, reviewer 3 asks about neuronal mitochondrial OxPhos. Given that mitochondrial morphology is an indirect readout of OxPhos, we performed *ex-vivo* whole brain metabolic analysis to measure mitochondrial oxygen consumption rate (OCR), a direct readout of mitochondrial oxidative phosphorylation (Figure 1B).

***Figure 1C:*** HSD treatment attenuates glycolysis in the brain.

- **Reviewer 2**
  - In comment #5, reviewer 2 has suggested that reduced Ldh expression is not sufficient to indicate attenuated glycolysis. The ex-vivo whole brain metabolic analysis displayed in Figure 1C revealed reduced extracellular flux acidification rate (ECAR) which is a direct readout of glycolysis.

***Figure 1B-C:*** HSD causes a shift in brain metabolism from glycolysis to mitochondrial oxidative phosphorylation. This data addresses the following comments:

- **Reviewer 2**
  - In comment #9, reviewer 2 has raised concerns that the manuscript relies mainly on cytological approaches. In the revised manuscript, we performed a sophisticated *ex vivo* whole brain metabolic analysis using Agilent Seahorse (Figure 1B-C).

***Figure 1H:*** HSD causes an upregulation in glial lipid droplets.

- **Reviewer 1 (comment #1), Reviewer 2 (comment #7), Reviewer 3 (comment #2)**
  - All reviewers requested quantitative comparison of lipid droplet levels between ND and HSD-fed flies. To address this, we performed lipid content analysis (Figure 1G) and quantified it as per reviewer request (Figure 1H).

***Figure S1:*** 2 weeks of HSD treatment does not alter Ldh levels.

- **Reviewer 1**
  - In comment #3, reviewer 1 asks us to address the specific effects of high sugar diet versus increased caloric intake. We find that subjecting the flies to a shorter HSD treatment does not alter Ldh levels despite a presumably increased caloric intake compared to ND-fed flies. We note the 2 weeks’ time point has been previously determined by us to be the point at which metabolic collapse occurs(16)
- **Reviewer 2**
  - In comment #3, reviewer 2 asks whether we see a similar phenotype with HFD. Given that it takes >2 weeks of HSD treatment to observe metabolic changes in the brain, and prolonged HFD in flies is lethal. We are unable to investigate the effects of prolonged HFD on the brain’s metabolic state.

***Figure 2A:*** HSD upregulates the transcript levels of the insulin resistance marker *G6P*.

- **Reviewer 2**
  - In comment #1, reviewer 2 asks for additional markers of insulin resistance. We show that G6P levels which are inversely correlated with insulin sensitivity are elevated in HSD-fed fly brains.
  - In comment #9, reviewer 2 has raised concerns that the manuscript relies mainly on cytological approaches. Here, we employ qPCR methods in addition to the cytological/genetic methods we previously presented.

***Figure 2B:*** HSD upregulates the transcript levels of the insulin receptor substrate *chico* suggesting insulin resistance.

- **Reviewer 2**
  - In comment #1, reviewer 2 asks for additional markers of insulin resistance. We show that G6P levels are elevated, which normally occurs under low insulin signaling conditions indicating insulin resistance.
  - In comment #9, reviewer 2 has raised concerns that the manuscript relies mainly on cytological approaches. Here, we employ qPCR methods in addition to the cytological/genetic methods we previously presented.

***Figure 2B:*** HSD increases Dilp5 secretion.

- **Reviewer 1**
  - In comment #2, reviewer 1 points to the limitation of relying solely on Dilp5 immunofluorescence on interpreting Dilp5 secretion. We performed qPCR on ND and HSD-fed fly brains to measure *Dilp5* mRNA levels.
- **Reviewer 2**
  - In comment #9, reviewer 2 has raised concerns that the manuscript relies mainly on cytological approaches. Here, we employ qPCR methods in addition to the cytological/genetic methods we previously presented.

***Figure S3:*** 2 weeks of HSD treatment does not alter Draper levels.

- **Reviewer 1**
  - In comment #3, reviewer 1 asks us to address the specific effects of high sugar diet versus increased caloric intake. We find that subjecting the flies to a shorter HSD treatment does not alter Draper levels despite a presumably increased caloric intake compared to ND-fed flies. We note the 2 weeks time point has been previously determined by us to be the point at which metabolic collapse occurs(16)
- **Reviewer 2**
  - In comment #3, reviewer 2 asks whether we see a similar phenotype with HFD. Given that it takes >2 weeks of HSD treatment to observe metabolic changes in the brain, and prolonged HFD in flies is lethal. We are unable to investigate the effects of prolonged HFD on glial phagocytic function or Draper expression.

***Figure S4:*** prolonged HSD reduces Draper levels in cortex glia.

- **Reviewer 1 (comment #4) and Reviewer 3 (comment #1)**
  - The reviewers ask us whether HSD-induced Draper dysregulation is unique to ensheathing glia. We analyzed Draper levels in the cortex glia, which is the only other glial subtype that expresses Draper(23, 26, 29). We observe a significant downregulation in basal Draper levels similar to ensheathing glia.

***Video 1:*** HSD impairs glial clearance of axonal debris after injury.

- **Reviewer 1**
  - In comment #3, reviewer 1 suggests that we include a 3D video depicting the olfactory axons in the ND and HSD-fed flies post injury.

References

1.	Pasco MY, Leopold P. High sugar-induced insulin resistance in Drosophila relies on the lipocalin Neural Lazarillo. PLoS One. 2012;7(5):e36583.

2.	Geminard C, Rulifson EJ, Leopold P. Remote control of insulin secretion by fat cells in Drosophila. Cell Metab. 2009;10(3):199-207.

3.	Hakim-Mishnaevski K, Flint-Brodsly N, Shklyar B, Levy-Adam F, Kurant E. Glial Phagocytic Receptors Promote Neuronal Loss in Adult Drosophila Brain. Cell Rep. 2019;29(6):1438-48 e3.

4.	Freeman MR. Drosophila Central Nervous System Glia. Cold Spring Harb Perspect Biol. 2015;7(11).

5.	Raiders S, Han T, Scott-Hewitt N, Kucenas S, Lew D, Logan MA, et al. Engulfed by Glia: Glial Pruning in Development, Function, and Injury across Species. J Neurosci. 2021;41(5):823-33.

6.	Lee CY, Landreth GE. The role of microglia in amyloid clearance from the AD brain. J Neural Transm (Vienna). 2010;117(8):949-60.

7.	Pagliassotti MJ, Wei Y, Bizeau ME. Glucose-6-phosphatase activity is not suppressed but the mRNA level is increased by a sucrose-enriched meal in rats. J Nutr. 2003;133(1):32-7.

8.	Ortmeyer HK, Bodkin NL, Hansen BC. Relationship of skeletal muscle glucose 6-phosphate to glucose disposal rate and glycogen synthase activity in insulin-resistant and non-insulin-dependent diabetic rhesus monkeys. Diabetologia. 1994;37(2):127-33.

9.	Kim GY, Lee YM, Cho JH, Pan CJ, Jun HS, Springer DA, et al. Mice expressing reduced levels of hepatic glucose-6-phosphatase-alpha activity do not develop age-related insulin resistance or obesity. Hum Mol Genet. 2015;24(18):5115-25.

10.	Gardner LB, Liu Z, Barrett EJ. The role of glucose-6-phosphatase in the action of insulin on hepatic glucose production in the rat. Diabetes. 1993;42(11):1614-20.

11.	Na J, Musselman LP, Pendse J, Baranski TJ, Bodmer R, Ocorr K, et al. A Drosophila model of high sugar diet-induced cardiomyopathy. PLoS genetics. 2013;9(1):e1003175.

12.	Villanueva JE, Livelo C, Trujillo AS, Chandran S, Woodworth B, Andrade L, et al. Time-restricted feeding restores muscle function in Drosophila models of obesity and circadian-rhythm disruption. Nat Commun. 2019;10(1):2700.

13.	Gill S, Le HD, Melkani GC, Panda S. Time-restricted feeding attenuates age-related cardiac decline in Drosophila. Science. 2015;347(6227):1265-9.

14.	Mihaylova MM, Chaix A, Delibegovic M, Ramsey JJ, Bass J, Melkani G, et al. When a calorie is not just a calorie: Diet quality and timing as mediators of metabolism and healthy aging. Cell Metab. 2023;35(7):1114-31.

15.	Livelo C, Guo Y, Abou Daya F, Rajasekaran V, Varshney S, Le HD, et al. Time-restricted feeding promotes muscle function through purine cycle and AMPK signaling in Drosophila obesity models. Nat Commun. 2023;14(1):949.

16.	Kelly KP, Alassaf M, Sullivan CE, Brent AE, Goldberg ZH, Poling ME, et al. Fat body phospholipid state dictates hunger-driven feeding behavior. Elife. 2022;11.

17.	Musselman LP, Fink JL, Narzinski K, Ramachandran PV, Hathiramani SS, Cagan RL, et al. A high-sugar diet produces obesity and insulin resistance in wild-type Drosophila. Dis Model Mech. 2011;4(6):842-9.

18.	Neville KE, Bosse TL, Klekos M, Mills JF, Weicksel SE, Waters JS, et al. A novel ex vivo method for measuring whole brain metabolism in model systems. J Neurosci Methods. 2018;296:32-43.

19.	Stanhope BA, Jaggard JB, Gratton M, Brown EB, Keene AC. Sleep Regulates Glial Plasticity and Expression of the Engulfment Receptor Draper Following Neural Injury. Curr Biol. 2020;30(6):1092-101.e3.

20.	Hilu-Dadia R, Hakim-Mishnaevski K, Levy-Adam F, Kurant E. Draper-mediated JNK signaling is required for glial phagocytosis of apoptotic neurons during Drosophila metamorphosis. Glia. 2018;66(7):1520-32.

21.	Ray A, Speese SD, Logan MA. Glial Draper Rescues Abeta Toxicity in a Drosophila Model of Alzheimer's Disease. J Neurosci. 2017;37(49):11881-93.

22.	Lu TY, MacDonald JM, Neukomm LJ, Sheehan AE, Bradshaw R, Logan MA, et al. Axon degeneration induces glial responses through Draper-TRAF4-JNK signalling. Nat Commun. 2017;8:14355.

23.	Purice MD, Speese SD, Logan MA. Delayed glial clearance of degenerating axons in aged Drosophila is due to reduced PI3K/Draper activity. Nat Commun. 2016;7:12871.

24.	Musashe DT, Purice MD, Speese SD, Doherty J, Logan MA. Insulin-like Signaling Promotes Glial Phagocytic Clearance of Degenerating Axons through Regulation of Draper. Cell Rep. 2016;16(7):1838-50.

25.	Doherty J, Sheehan AE, Bradshaw R, Fox AN, Lu TY, Freeman MR. PI3K signaling and Stat92E converge to modulate glial responsiveness to axonal injury. PLoS Biol. 2014;12(11):e1001985.

26.	Doherty J, Logan MA, Tasdemir OE, Freeman MR. Ensheathing glia function as phagocytes in the adult Drosophila brain. J Neurosci. 2009;29(15):4768-81.

27.	MacDonald JM, Beach MG, Porpiglia E, Sheehan AE, Watts RJ, Freeman MR. The Drosophila cell corpse engulfment receptor Draper mediates glial clearance of severed axons. Neuron. 2006;50(6):869-81.

28.	Bailey AP, Koster G, Guillermier C, Hirst EM, MacRae JI, Lechene CP, et al. Antioxidant Role for Lipid Droplets in a Stem Cell Niche of Drosophila. Cell. 2015;163(2):340-53.

29.	Nakano R, Iwamura M, Obikawa A, Togane Y, Hara Y, Fukuhara T, et al. Cortex glia clear dead young neurons via Drpr/dCed-6/Shark and Crk/Mbc/dCed-12 signaling pathways in the developing Drosophila optic lobe. Dev Biol. 2019;453(1):68-85.
